# Supplementary material for: Associations of hurricane exposure and forecasting with impaired birth outcomes
Source: Nat Commun. 2022 Nov 8;13:6746. doi: 10.1038/s41467-022-33865-x (PMC9643368; doi:10.1038/s41467-022-33865-x)
Supplement: Supplementary file 1 — Supplementary Information [file 41467_2022_33865_MOESM1_ESM.pdf]

Supplementary Information

Expecting Mother Nature and the Disruptive Birth Impacts of Hurricanes and Their Forecasts

Author list:

Jacob Hochard[1*], Yuanhao Li[2] and Nino Abashidze[1]

Author affiliations:

[1]Haub School of Environment and Natural Resources, University of Wyoming, Bim Kendall House, 804 E Fremont Street, Laramie, WY 82072, USA

[2]SNF - Centre for Applied Research at NHH, Helleveien 30, N-5045, Bergen, Norway

Corresponding author:

*To whom correspondence should be addressed; E-mail: JHochard@uwyo.edu.

Supplementary Table 1: Full sample summary statistics (inclusive of treatment and control groups).

Panel A: Descriptive Statistics

Variable	Mean	Std. Dev.	Min.	Max.	N
Birth weight (g)	3263	601	28	6435	710186
Gestation length (wk)	38.54	2.13	23	45	710310
Month prenatal care began	2.6	1.447	0	9	582407
Number of prenatal care visits	12.21	4.01	0	49	702336
One-day max rainfall	1.60	2.11	0	10.21	709613
Number of 6-hour advisories	9.27	4.64	0	22	710310

Panel B: Frequencies of binary variables

Variable	0	1	N
Low birth weight (<2500g)	648513	61797	710310
Very low birth weight (<1500g)	699655	10655	710310
Preterm (< 37 wks)	637858	72452	710310
Extreme preterm (< 34 wks)	689711	20599	710310

Notes: Source data are provided as a Source Data file.

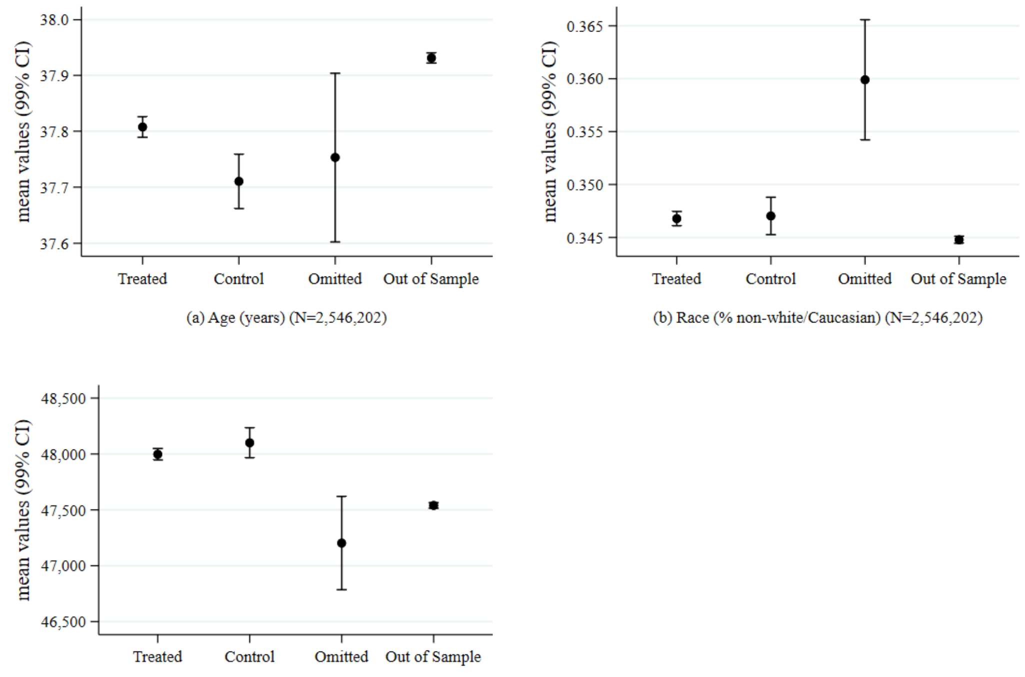

(a) Age (years) (N=2,546,202)

(b) Race (% non-white/Caucasian) (N=2,546,202)

(c) Income (USD) (N=2,546,202)

Supplementary Figure 1: Zip code-level comparisons of median female age (2011 American Community Survey 5-year estimate), median non-white proportion (2011 decennial American Community Survey 5-year estimate) and median household income (2011 American Community Survey 5-year estimate) weighted by observation frequency. In-sample observations include the treated and control groups. The omitted group contains North Carolina residents who were pregnant during the sample window but excluded from the sample based on selection-into-sample criteria. The out of sample group includes North Carolina residents who gave birth from 1/1/1996 to 8/25/2006 and 6/5/2012 to 12/31/2017. 99% confidence intervals are presented and the vertical axis is condensed to highlight these error bands. Source data are provided as a Source Data file.

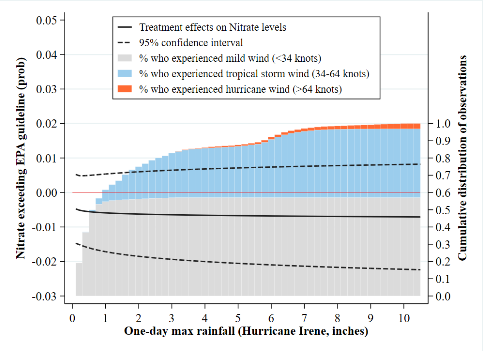

(a) Nitrate (N=10,702)

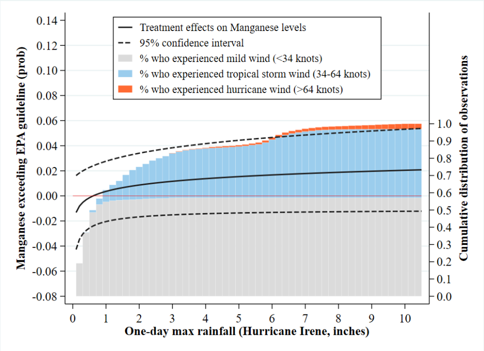

(b) Manganese (N=16,395)

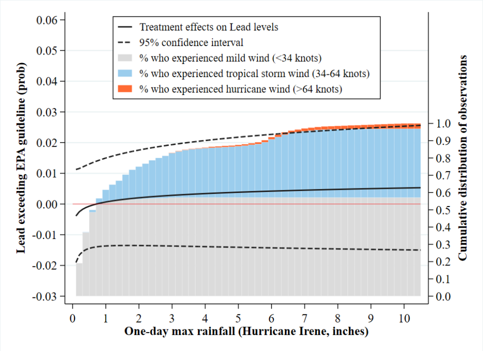

(c) Lead (N=17,105)

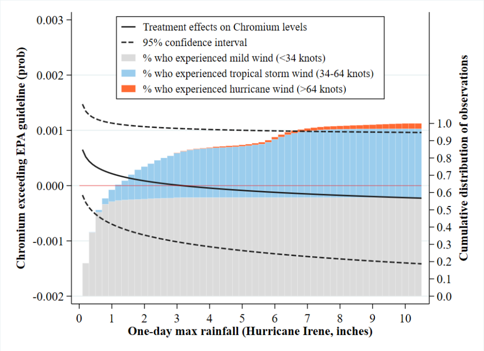

(d) Chromium (N=16,382)

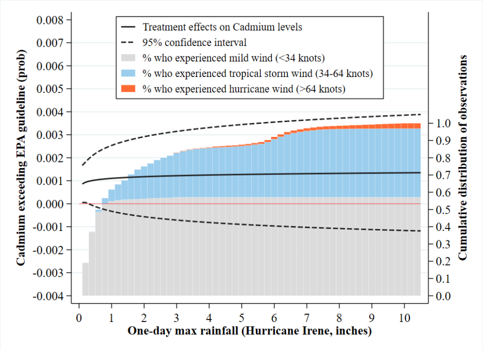

(e) Cadmium (N=16,425)

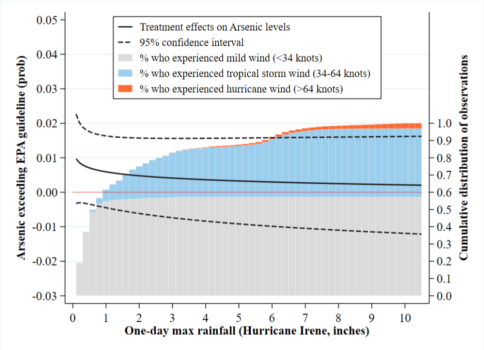

(f) Arsenic (N=16,435)

Supplementary Figure 2: Estimated treatment effect of Hurricane Irene exposure on the likelihood of a private well water sample exceeding EPA guidelines for nitrate, manganese, lead, chromium, cadmium and arenic. Rainfall at residential address where well water sample was taken was used as an indicator of exposure intensity represented by the one-day maximum rainfall from August 14, 2011 to September 4, 2011, which encompassed the hurricane event's impact on North Carolina. While well water samples were not taken from the same residences as our pregnant women sample, the same selection into treatment criteria was used for sampling date, rather than conception date. Estimated treatment effects were overlayed with a cumulative distribution of wind speed exposures. Rainfall source: Parameter-elevation Regressions on Independent Slopes Model (PRISM) climate group Time Series Values for Individual Locations. Source data are provided as a Source Data file.

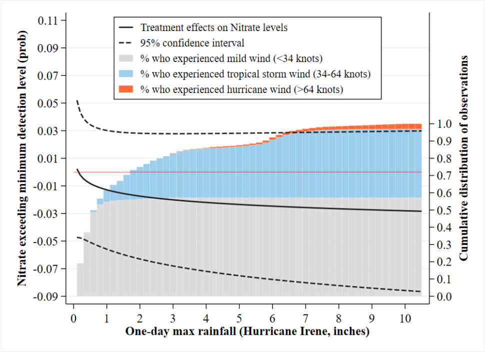

(a) Nitrate (N=10,702)

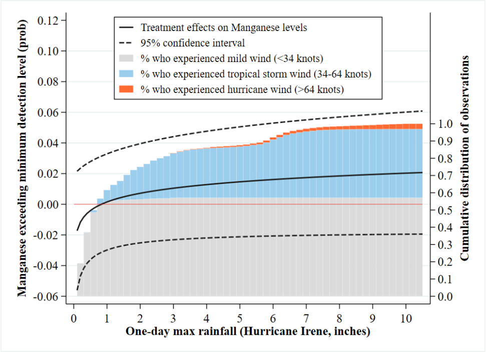

(b) Manganese (N=16,395)

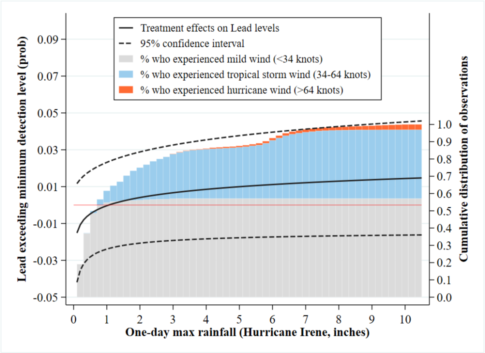

(c) Lead (N=17,105)

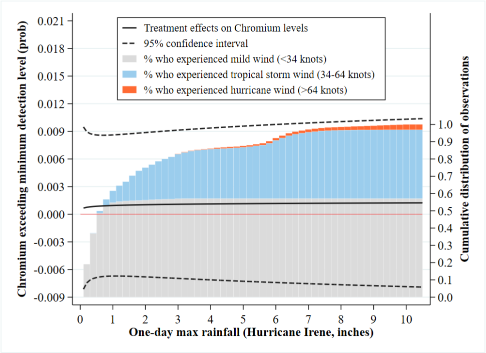

(d) Chromium (N=16,382)

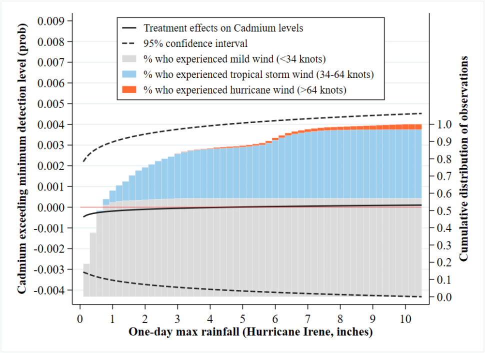

(e) Cadmium (N=16,425)

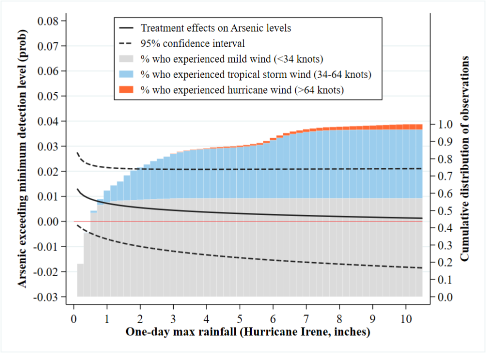

(f) Arsenic (N=16,435)

Supplementary Figure 3: Estimated treatment effect of Hurricane Irene exposure on the likelihood of a private well water sample exceeding the North Carolina State Laboratory's minimum detection limit. Rainfall at residential address where well water sample was taken was used as an indicator of exposure intensity represented by the one-day maximum rainfall from August 14, 2011 to September 4, 2011, which encompassed the hurricane event's impact on North Carolina. While well water samples were not taken from the same residences as our pregnant women sample, the same selection into treatment criteria was used for sampling date, rather than conception date. Estimated treatment effects were overlayed with a cumulative distribution of wind speed exposures. Rainfall source: Parameter-elevation Regressions on Independent Slopes Model (PRISM) climate group Time Series Values for Individual Locations. Source data are provided as a Source Data file.

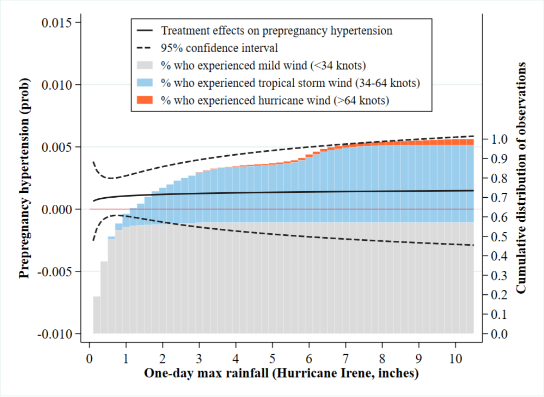

(a) Diagnosed with prepregnancy hypertension

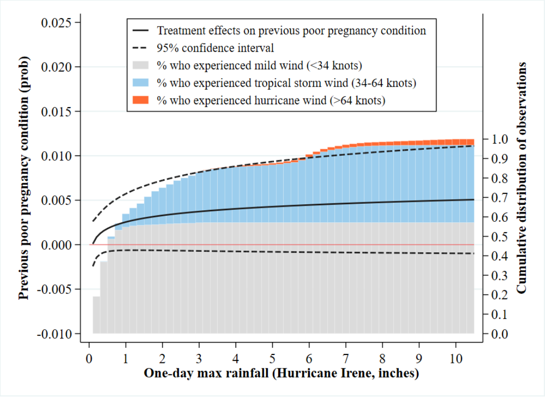

(b) Previously experienced a poor pregnancy outcome

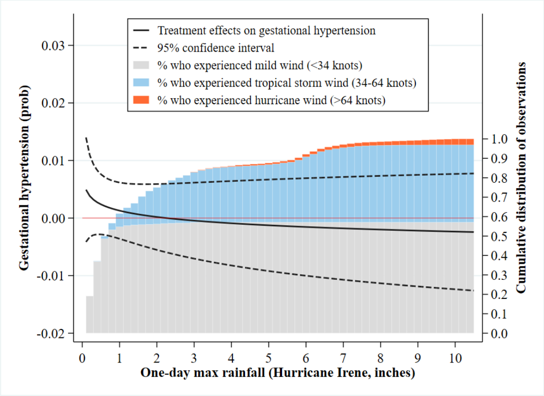

(c) Diagnosed with gestational hypertension

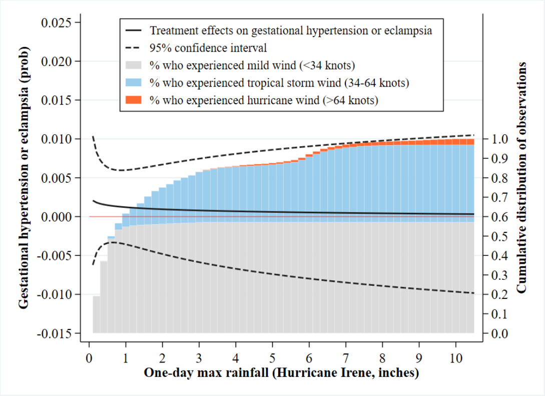

(d) Diagnosed with gestational hypertension or eclampsia

Supplementary Figure 4: Estimated treatment effect of Hurricane Irene exposure on the likelihood of being diagnosed with various medical risk factors (prepregnancy hypertension, previous poor pregnancy, gestational hypertension or gestational hypertension/eclampsia. Rainfall at residential address where well water sample was taken was used as an indicator of exposure intensity represented by the one-day maximum rainfall from August 14, 2011 to September 4, 2011, which encompassed the hurricane event's impact on North Carolina. Estimated treatment effects were overlayed with a cumulative distribution of wind speed exposures. Rainfall source: Parameter-elevation Regressions on Independent Slopes Model (PRISM) climate group Time Series Values for Individual Locations. Source data are provided as a Source Data file.
