# Supplementary material for: Associations of hurricane exposure and forecasting with impaired birth outcomes
Source: Nat Commun. 2022 Nov 8;13:6746. doi: 10.1038/s41467-022-33865-x (PMC9643368; doi:10.1038/s41467-022-33865-x)
Supplement: Supplementary file 2 — Reporting Summary [file 41467_2022_33865_MOESM2_ESM.pdf]

nature portfolio

Corresponding author(s): Jacob Hochard

Last updated by author(s): Aug 7, 2022

Reporting Summary

Nature Portfolio wishes to improve the reproducibility of the work that we publish. This form provides structure for consistency and transparency in reporting. For further information on Nature Portfolio policies, see our Editorial Policies and the Editorial Policy Checklist.

Statistics

For all statistical analyses, confirm that the following items are present in the figure legend, table legend, main text, or Methods section.

n/a	Confirmed	
☐	☒	The exact sample size (n) for each experimental group/condition, given as a discrete number and unit of measurement
☐	☒	A statement on whether measurements were taken from distinct samples or whether the same sample was measured repeatedly
☐	☒	The statistical test(s) used AND whether they are one- or two-sided<br>*Only common tests should be described solely by name; describe more complex techniques in the Methods section.*
☐	☒	A description of all covariates tested
☒	☐	A description of any assumptions or corrections, such as tests of normality and adjustment for multiple comparisons
☐	☒	A full description of the statistical parameters including central tendency (e.g. means) or other basic estimates (e.g. regression coefficient) AND variation (e.g. standard deviation) or associated estimates of uncertainty (e.g. confidence intervals)
☒	☐	For null hypothesis testing, the test statistic (e.g. F, t, r) with confidence intervals, effect sizes, degrees of freedom and P value noted<br>*Give P values as exact values whenever suitable.*
☒	☐	For Bayesian analysis, information on the choice of priors and Markov chain Monte Carlo settings
☒	☐	For hierarchical and complex designs, identification of the appropriate level for tests and full reporting of outcomes
☒	☐	Estimates of effect sizes (e.g. Cohen's d, Pearson's r), indicating how they were calculated

Our web collection on statistics for biologists contains articles on many of the points above.

Software and code

Policy information about availability of computer code

Data collection	no software was used for data collection.
Data analysis	All econometric analyses were conducted using Stata/MP 16.1 and geospatial analysis was completed in ArcGIS 10.8.

For manuscripts utilizing custom algorithms or software that are central to the research but not yet described in published literature, software must be made available to editors and reviewers. We strongly encourage code deposition in a community repository (e.g. GitHub). See the Nature Portfolio guidelines for submitting code & software for further information.

Data

Policy information about availability of data

All manuscripts must include a data availability statement. This statement should provide the following information, where applicable:

- Accession codes, unique identifiers, or web links for publicly available datasets
- A description of any restrictions on data availability
- For clinical datasets or third party data, please ensure that the statement adheres to our policy

The data used in this analysis contain sensitive information with individual-level health identifiers. An anonymized dataset for replication will be released in accordance with our data use agreement and upon request (send requests to JHochard@uwyo.edu).

Field-specific reporting

Please select the one below that is the best fit for your research. If you are not sure, read the appropriate sections before making your selection.

☐ Life sciences ☒ Behavioural & social sciences ☐ Ecological, evolutionary & environmental sciences

For a reference copy of the document with all sections, see nature.com/documents/nr-reporting-summary-flat.pdf

Behavioural & social sciences study design

All studies must disclose on these points even when the disclosure is negative.

Study description	The quantitative data are used in this study.
Research sample	The data include all (N=710,186) North Carolina birth outcomes that are in the path of Hurricane Irene. Data are obtained from North Carolina Department of Health and Human Services for all North Carolina live and still births for our study period (from August 26th, 2006 to June 14th, 2012). The data include birth date, birth outcomes (birth weight and gestation length), mothers' residential addresses as well as prenatal care and medical risk factor information.
Sampling strategy	The analyzed data set includes all officially recorded births in North Carolina for our study area and study period.
Data collection	After we obtained the IRB approval for this study (the study is governed by East Carolina University's IRB 17-000354), we contacted the North Carolina Department of Health and Human Services and obtained all North Carolina live and still births for out study period. The North Carolina Department of Health and Human Services was blinded to study hypothesis.
Timing	The data were received in April 2020.
Data exclusions	All births that did not experience pre-natal or post-natal exposure to Hurricane Irene were excluded from the analyses.
Non-participation	No participants were involved in the study.
Randomization	All pre-hurricane births served as a control group for post-hurricane births within the same communities that define our treatment group.

Reporting for specific materials, systems and methods

We require information from authors about some types of materials, experimental systems and methods used in many studies. Here, indicate whether each material, system or method listed is relevant to your study. If you are not sure if a list item applies to your research, read the appropriate section before selecting a response.

Materials & experimental systems

n/a	Involved in the study	
☒	☐	Antibodies
☒	☐	Eukaryotic cell lines
☒	☐	Palaeontology and archaeology
☒	☐	Animals and other organisms
☐	☒	Human research participants
☒	☐	Clinical data
☒	☐	Dual use research of concern

Methods

n/a	Involved in the study	
☒	☐	ChIP-seq
☒	☐	Flow cytometry
☒	☐	MRI-based neuroimaging

Human research participants

Policy information about studies involving human research participants

Population characteristics	Data do not include information on age , gender, or race of participants.
Recruitment	Sample includes all recorded births in North Carolina for our study area and study period.
Ethics oversight	The study is governed by East Carolina University's IRB 17-000354. The written informed consents for all the data were waived by the corresponding IRB.

Note that full information on the approval of the study protocol must also be provided in the manuscript.
